# Psychometric Evaluation of the German Version of the Demoralization Scale-II and the Association Between Demoralization, Sociodemographic, Disease- and Treatment-Related Factors in Patients With Cancer

**DOI:** 10.3389/fpsyg.2021.789793

**Published:** 2021-11-24

**Authors:** Susan Koranyi, Andreas Hinz, Julia M. Hufeld, Tim J. Hartung, Leonhard Quintero Garzón, Uta Fendel, Anne Letsch, Matthias Rose, Peter Esser, Anja Mehnert-Theuerkauf

**Affiliations:** ^1^Department of Medical Psychology and Medical Sociology, University Medical Center Leipzig, Leipzig, Germany; ^2^Department of Neurology, Charité - Universitätsmedizin Berlin, Corporate Member of Freie Universität Berlin, Humboldt-Universität zu Berlin, Berlin, Germany; ^3^Department of Psychosomatic Medicine, Charité - Universitätsmedizin Berlin, Corporate Member of Freie Universität Berlin, Humboldt-Universität zu Berlin, Berlin, Germany; ^4^Department of Hematology and Oncology, Campus Benjamin Franklin, Charité -Universitätsmedizin Berlin, Corporate Member of Freie Universität Berlin, Humboldt-Universität zu Berlin, and Berlin Institute of Health, Berlin, Germany; ^5^Department of Medicine II, Hematology and Oncology, University Hospital Schleswig-Holstein, Kiel, Germany; ^6^Department of Quantitative Health Sciences, Medical School, University of Massachusetts, Amherst, MA, United States

**Keywords:** psychometrics, validation study, confirmatory factor analysis, demoralization, neoplasm

## Abstract

**Objective:** To test the psychometric properties, internal consistency, dimensional structure, and convergent validity of the German version of the Demoralization Scale-II (DS-II), and to examine the association between demoralization, sociodemographic, disease- and treatment-related variables in patients with cancer.

**Methods:** We recruited adult patients with cancer at a Psychosocial Counseling Center and at oncological wards. Participants completed the 16-item DS-II, Patient Health Questionnaire-9 (PHQ-9), Generalized Anxiety Disorder Screener-2 (GAD-2), Distress Thermometer (DT), and Body Image Scale (BIS). We analyzed internal consistency of the DS-II using Cronbach‘s Alpha (α). We tested the dimensional structure of the DS-II with Confirmatory Factor Analyses (CFA). Convergent validity was expressed through correlation coefficients with established measures of psychological distress. The associations between demoralization, sociodemographic, disease- and treatment-related variables were examined with ANOVAs.

**Results:** Out of 942 eligible patients, 620 participated. The average DS-II total score was *M* = 5.78, *SD* = 6.34, the *Meaning and Purpose* subscale *M* = 2.20, *SD* = 3.20, and the *Distress and Coping Ability* subscale *M* = 3.58, *SD* = 3.45. Internal consistency ranged from high to excellent with α = 0.93 for the DS-II total scale, α = 0.90 for the *Meaning and Purpose* subscale, and α = 0.87 for the *Distress and Coping Ability* subscale. The one-factor and the two-factor model yielded similar model fits, with CFI and TLI ranging between 0.910 and 0.933, SRMR < 0.05. The DS-II correlated significantly with depression (PHQ-9: *r* = 0.69), anxiety (GAD-2: *r* = 0.72), mental distress (DT: *r* = 0.36), and body image disturbance (BIS: *r* = 0.58). High levels of demoralization were reported by patients aged between 18 and 49 years (*M* = 7.77, *SD* = 6.26), patients who were divorced/separated (*M* = 7.64, *SD* = 7.29), lung cancer patients (*M* = 9.29, *SD* = 8.20), and those receiving no radiotherapy (*M* = 7.46, *SD* = 6.60).

**Conclusion:** The DS-II has very good psychometric properties and can be recommended as a reliable tool for assessing demoralization in patients with cancer. The results support the implementation of a screening for demoralization in specific risk groups due to significantly increased demoralization scores.

## Introduction

Cancer diagnosis and treatment are frequently associated with psychological distress (Hartung et al., 2017; Mehnert et al., 2018; Pitman et al., 2018; Carlson et al., 2019). Considerable research has shown a wide spectrum of psychological states ranging from subsyndromal psychological distress and mental disorders (Mehnert et al., 2014; Ernst et al., 2021) to existential distress such as demoralization (Robinson et al., 2015; Tang et al., 2015; Robinson et al., 2016c). Prevalence rates of demoralization syndrome range between 13 and 33% depending on the method of measurement used and characteristics of the patient sample (Robinson et al., 2015).

Based on the work of de Figueiredo and Frank (de Figueiredo and Frank, 1982), among others, Kissane and Clark (Kissane et al., 2001) conceptualized demoralization as a state of maladaptive coping characterized by a loss of purpose and meaning in life, low morale, low optimism, as well as helplessness and hopelessness (Clarke and Kissane, 2002; Kissane, 2014; Robinson et al., 2015). Demoralization can influence mood and the ability to cope with threatening life events (Clarke and Kissane, 2002); it also negatively influences social functioning, decision making, and quality of life (Nanni et al., 2018), and causes a feeling of dependence and being a burden on others (Robinson et al., 2015; Vehling et al., 2017). Demoralization is a common presentation of existential distress and is considered a significant mental health concern since it is accompanied with the desire for hastened death and is a risk factor for suicide (Vehling et al., 2017; Nanni et al., 2018; McFarland et al., 2019; Rzeszut and Assael, 2021). It also causes problems in building sustainable and trusting relationships with health care providers (Quintero Garzón et al., 2018) and family caregivers (Bovero et al., 2021).

Kissane and colleagues developed the Demoralization Scale (DS-I; Kissane et al., 2004), a 24-item scale with a five-factorial structure and response options ranging from 0 (“never”) to 4 (“all the time”). The DS-I has been adapted to several languages (Mullane et al., 2009; Hung et al., 2010; Mehnert et al., 2011; Costantini et al., 2013; Grassi et al., 2017), and has been found to have acceptable to excellent psychometric properties. The German version of the DS-I has high internal consistency (α = 0.84) for the total score and good construct validity with other mental health-related measures (Mehnert et al., 2011). Applications of the DS-I have been performed in several oncological studies (Robinson et al., 2015, 2016c).

The DS-II, a more recently developed revised short-version of the DS-I, is a 16-item version of the Demoralization Scale (Robinson et al., 2016a,b) that has fewer items and answer options (three instead of five) making it more user-friendly for patients with advanced cancer and patients in the palliative care period. Initial results for the DS-II scale’s reliability and validity have been published based on an Australian sample of 211 patients receiving palliative care, most of them suffering from cancer (Robinson et al., 2016b). The DS-II demonstrated high internal consistency (α = 0.89 for the total scale; α = 0.84 for the *Meaning and Purpose* subscale, and α = 0.82 for the *Distress and Coping Ability* subscale) (Robinson et al., 2016b). Subsequently, a Spanish version of the DS-II was tested in a sample of 150 patients with advanced cancer stages in Spain and a number of Latin American countries (Belar et al., 2019). The Spanish DS-II version also showed a high internal consistency with α = 0.88 (total DS-II scale), α = 0.83 (*Meaning and Purpose)*, and α = 0.79 (*Distress and Coping Ability*) (Belar et al., 2019). Recently, a Chinese version of the DS-II was used in a cross-sectional study to assess demoralization in Chinese patients with advanced cancer (Wu et al., 2021). The internal reliability of the Chinese DS-II was excellent, with α = 0.91 for the total scale, and α = 0.91 and α = 0.89 for its two subscales (Wu et al., 2021). However, although the DS-II had already been translated and used in various languages, it was not until the present study was conducted that it was translated into German or a psychometric evaluation of that version was carried out. Furthermore, none of the previously conducted studies replicated the proposed two-factor solution of the original DS-II scale (Robinson et al., 2016b) using Confirmatory Factor Analyses.

Previous studies consistently reported a strong positive relationship between demoralization and depression (Katz et al., 2001; Grassi et al., 2004; Kissane et al., 2004; Mullane et al., 2009; Mehnert et al., 2011; Hadnagy et al., 2012; Lee et al., 2012; Vehling et al., 2012, 2013; Costantini et al., 2013) and demoralization and anxiety (Katz et al., 2001; Grandi et al., 2011; Mehnert et al., 2011; Vehling et al., 2011). Recently, the short version of the DS-II scale has also been used to investigate the association between demoralization and other established measures of psychological distress. For example, a strong positive association between DS-II and depression scores were reported (Ignatius and De La Garza, 2019; Wu et al., 2021). Furthermore, various studies across different cancer populations have demonstrated strong associations between depression and body image disturbance, an issue which is known to greatly burden cancer survivors suffering from severe disease- and treatment-related body changes (e.g., body disfigurement, skin changes, weight gain or loss, scars, and hair loss) (Hartung et al., 2021). However, no previous study had investigated whether body image disturbance also goes along with feelings of demoralization.

The relationship between demoralization and disease- and treatment-related factors was frequently studied in previous studies, consistently revealing no association between time since diagnosis (Katz et al., 2001; Boscaglia and Clarke, 2007; Mehnert et al., 2011; Robinson et al., 2016a), stage of disease (Grassi et al., 2004; Boscaglia and Clarke, 2007; Lee et al., 2012; Vehling et al., 2012; Robinson et al., 2016a), type of treatment (Grassi et al., 2004; Vehling et al., 2012), and demoralization scores. However, inconsistent findings were reported with regard to the association between tumor site and demoralization. One study recorded higher demoralization scores in patients with head and neck cancer compared to patients with cervical or gastrointestinal cancer (Lee et al., 2012), whereas other studies found no association (Mehnert et al., 2011; Robinson et al., 2016a).

Regarding sociodemographic factors, consistent evidence was reported for the association between employment and demoralization, with lower demoralization reported by employed patients in comparison to jobless patients (Katz et al., 2001; Lee et al., 2012). Similar results for the relationship between marital status and demoralization were reported by three studies, revealing that living with a partner was associated with lower levels of demoralization (Katz et al., 2001; Grandi et al., 2011; Mehnert et al., 2011). However, mixed evidence was obtained regarding age, gender, and education. In two studies, demoralization was unrelated to age (Katz et al., 2001; Quintero Garzón et al., 2021), whereas, another study demonstrated that older age was related to higher scores of demoralization (Vehling et al., 2011). In contrast, two studies reported a negative correlation between age and demoralization (Mehnert et al., 2011; Vehling et al., 2013). There are also no consistent findings with regard to gender-specific differences in demoralization scores. While some studies indicated that women report higher demoralization scores (Grandi et al., 2011; Mehnert et al., 2011; Vehling et al., 2011, 2013), two other studies reported no gender differences (Grassi et al., 2005; Lee et al., 2012). Additionally, previous studies found no consistent association between education and demoralization (Katz et al., 2001; Lee et al., 2012).

Based on previous evidence, the aims of this study were (1) to examine psychometric properties of the German version of the DS-II scale and to calculate internal consistency in a sample of patients with cancer; (2) to test the two-factor structure of DS-II scale; (3) to verify convergent validity of DS-II scale with established measures of depression, anxiety, mental distress, and body image disturbance; and (4) to determine the relationship between demoralization and sociodemographic and disease- and treatment-related factors.

## Materials and Methods

### Patient Sample

Participants were adult (≥18 years) patients with a confirmed diagnosis of any cancer type (ICD-10 code C00 - C97) who were recruited consecutively from two study centers, one in Leipzig and the other in Berlin, Germany, between May 2018 and January 2020. In the Leipzig study center, the patients were recruited from the Psychosocial Counseling Center for patients with cancer at the Leipzig University Hospital. In Berlin, the patients were recruited from the wards of two departments, the Department of Radiation Oncology and Radiotherapy, and the Department of Hematology and Oncology, of the Charité University Medical Center (Hartung et al., 2021). After having the aims of the study explained to them, all participants gave informed consent in accordance to the Declaration of Helsinki. Patients were not included in the study if they were not proficient in German or if they were pregnant or breastfeeding. The study was approved by the Ethics Committee of the University of Leipzig (reference number 090/18-ek).

### Measures

We collected sociodemographic and disease- and treatment-related variables *via* standardized patient self-report questionnaires.

#### Demoralization

The Demoralization Scale DS-II is an established and validated self-report questionnaire with 16 items rated using a three-point Likert scale (0 = “never”; 1 = “sometimes”; 2 = “often”), resulting in a total score ranging from 0 to 32. The DS-II contains two 8-item subscales called *Meaning and Purpose* and *Distress and Coping Ability* (see Table 2 for example of items). High scores represent high levels of demoralization (Robinson et al., 2016b). The authors of the original DS-II scale (Robinson et al., 2016b) defined the following cutoff criteria for the total score: low (0–3), moderate (4–10), and high (≥11) demoralization. We translated the DS-II scale from English to German according to state-of-the-art translation procedures (Sousa and Rojjanasrirat, 2011). First, two translators independently performed one German translation each. They then discussed their translations and agreed upon one version. Second, this German version was translated back into English by two other translators who did not know the original English version, and agreed on one final back translation. Third, this back translation was compared with the original questionnaire by an expert in psychosocial oncology. Differences were discussed in the research team, and consensus was reached upon a final German translation.

#### Depression

The Patient Health Questionnaire-9 (PHQ-9) is a 9-item instrument for measuring depression (Kroenke et al., 2001). Patients rate the symptoms they have experienced over the previous 2 weeks on a four-point Likert scale from 0 = “not at all” to 3 = “almost every day.” The sum score ranges from 0 to 27, and higher scores represent higher depressive symptomatology. The German version of PHQ-9 shows good internal consistencies, with α = 0.89 (Kroenke et al., 2001).

#### Anxiety

The Generalized Anxiety Disorder Screener-2 (GAD-2) is an ultra-short instrument for measuring anxiety experienced over the past 2 weeks (Löwe et al., 2010). Patients rate their symptoms on a four-point Likert scale from 0 = “not at all” to 3 = “almost every day,” resulting in a sum score ranging from 0 to 6. Higher scores indicate higher levels of anxiety. Cronbach‘s alpha = 0.72 demonstrates acceptable internal consistency (Hinz et al., 2017).

#### Mental Distress

The NCCN Distress Thermometer (DT) assesses patients’ overall psychological symptom burden during the past week on a visual analog scale (0–10), with higher scores representing higher mental distress (Mehnert et al., 2006).

#### Body Image Disturbance

The 10-item Body Image Scale (BIS) is a reliable and validated self-report scale to measure body image disturbance (Hopwood et al., 2001). Patients rate how they felt about their appearance during the past week on a four-point Likert scale ranging from 0 = “not at all” to 3 = “very much.” Sum scores range from 0 to 30, with higher scores indicating increased cancer-specific aspects of body image disturbance (e.g., worries about physical and sexual attractiveness and feelings of being permanently disfigured by surgical treatment). The German version of the BIS shows excellent internal consistency with α = 0.92 (Hartung et al., 2021).

### Statistical Analysis

The statistical procedures were performed with IBM SPSS, version 25; the Mplus 6.1 version was used for the Confirmatory Factor Analyses.

#### Missing Values

Patients were included in the statistical calculations if at least 75% of the items were valid on both subscales of the DS-II. In these cases, the missing values were replaced with the rounded mean of the valid items. If on at least one subscale the number of missing items was higher than 25%, the patients were excluded from further analysis.

#### Internal Consistency and Dimensional Structure of the Demoralization Scale-II

Internal consistency of the DS-II scale and its subscales was assessed with Cronbach’s alpha coefficients (α). Confirmatory Factor Analyses were performed to evaluate whether the proposed two-factor solution of the original DS-II scale (Robinson et al., 2016b) can be replicated with our sample of patients with cancer. Two models were tested: the one-factor model, aggregating across all 16 items, and the two-factor model according to the dimensional structure of two proposed subscales. The goodness-of-fit was evaluated using the Comparative Fit Index (CFI) and the Tucker Lewis Index (TLI) with cutoff values of >0.95 for “good” and >0.90 for an “acceptable” model fit. Badness-of-fit was estimated *via* Standardized Root Mean Square Residual (SRMR < 0.05 “good,” <0.10 “acceptable” fit) and the Root Mean Square Error of Approximation (RMSEA <0.06 “good,” <0.08 “acceptable” fit) (Hu and Bentler, 1999; Schermelleh-Engel et al., 2003).

#### Convergent Validity

To assess convergent validity with established measures of psychological distress, we calculated Pearson correlations coefficients between DS-II and PHQ-9 (depression), GAD-2 (anxiety), mental distress (DT) and body image disturbance (BIS). The threshold for convergent validity was determined with *r* ≥ 0.5 (Cohen, 1977).

#### Associations Between Demoralization, Sociodemographic, Disease- and Treatment-Related Variables

The relationship between demoralization and sociodemographic (age, gender, employment status, civil status, and education), and disease- and treatment related variables (tumor site, metastases, surgery, chemotherapy, radiotherapy, and hormone therapy) were tested with ANOVAs with the covariables *gender* and *age group*.

## Results

### Sample Characteristics

A total of 942 patients with cancer were eligible for the study, and 677 (response rate: 72%) were willing to participate. Ultimately, 620 patients (n__*counseling center*_ = 571, 92%; n__*oncological wards*_ = 49, 8%) provided valid data sets (age: *M* = 60.3 years; 57.7% women). Further sample characteristics are given in Table 1.

**TABLE 1:** Sample characteristics.

	Total (*N* = 620)		Men (*N* = 268)		Women (*N* = 352)	
	*N*	%	*N*	%	*N*	%
**Age, M *(SD)* [years]**	60.3	(13.4)	63.7	(12.4)	57.7	(13.6)
≤49 years	133	21.5	35	13.1	98	27.8
50 – 59 years	144	23.2	57	21.3	87	24.7
60 – 69 years	172	27.7	77	28.7	95	27.0
≥70 years	171	27.6	99	36.9	72	20.5
**Civil status ^(a)^**						
Single	97	15.8	39	14.7	58	16.6
Married	372	60.5	189	71.1	183	52.4
Divorced/separated	97	15.8	29	10.9	68	19.5
Widowed	49	8.0	9	3.4	40	11.5
**Education ^(a)^**						
≤10 years of education	367	60.1	165	62.7	202	58.0
≥11 years of education	244	39.9	98	37.3	146	42.0
**Employment status ^(a)^**						
Occupied	239	40.1	78	30.1	161	47.8
Unemployed	38	6.4	17	6.6	21	6.2
Retired	290	48.7	153	59.1	137	40.7
Other	29	4.9	11	4.2	18	5.3
**Tumor site**						
Breast	208	33.5	2	0.7	206	58.5
Prostate	104	16.8	104	38.8	0	0
Head and neck	51	8.2	36	13.4	15	4.3
Skin	38	6.1	23	8.6	15	4.3
Female genital organs	34	5.5	0	0.0	34	9.7
Hematological	32	5.2	15	5.6	17	4.8
Central nervous system	30	4.8	16	6.0	14	4.0
Lung	28	4.5	16	6.0	12	3.4
Colorectal	22	3.5	13	4.9	9	2.6
Other	73	11.8	43	16.0	30	8.5
**Metastases ^(a)^**						
No	477	81.3	201	78.8	276	83.1
Yes	110	18.7	54	21.2	56	16.9
Surgery ^(a)^						
No	140	23.1	95	36.3	45	13.1
Yes	465	76.9	167	63.7	298	86.9
**Chemotherapy ^(a)^**						
No	366	60.5	171	65.3	195	56.9
Yes	239	39.5	91	34.7	148	43.1
**Radiotherapy ^(a)^**						
No	175	28.8	77	29.2	98	28.6
Yes	432	71.2	187	70.8	245	71.4
**Hormone therapy ^(a)^**						
No	505	83.5	224	85.5	281	81.9
Yes	100	16.5	38	14.5	62	18.1

*^(a)^ missing data not reported.*

### Internal Consistency and Psychometric Properties of the Demoralization Scale-II

The Cronbach‘s alpha coefficients were α = 0.93 for the total scale and between α = 0.87 (*Distress and Coping Ability*) and α = 0.90 (*Meaning and Purpose)* for the subscales.

Mean scores and standard deviations on a single item level are presented in Table 2. In addition, we calculated the proportion of the patients who responded to the items with at least “sometimes” (summarizing the response codes of 1 = “sometimes” and 2 = “often”), see Table 2. The items with the highest affirmation scores were “I feel distressed about what is happening to me” (*M* = 0.74, *SD* = 0.68, 61%) and “I feel irritable” (*M* = 0.63, *SD* = 0.66, 52%). The lowest affirmation was given to the items “I am not a worthwhile person” (*M* = 0.15, *SD* = 0.39, 14%) and “I would rather not be alive” (*M* = 0.10, *SD* = 0.35, 9%).

**TABLE 2:** Item characteristics of the Demoralization Scale-II (DS-II).

	Item/Scale	Subscale	*M*	*SD*	%	*r*_it_ (sub)	*r*_it_ (tot)
(1)	There is little value in what I can offer others.	1	0.32	0.53	29	0.67	0.68
(2)	My life seems to be pointless.	1	0.24	0.49	21	0.75	0.73
(3)	My role in life has been lost.	1	0.31	0.57	25	0.73	0.76
(4)	I no longer feel emotionally in control.	2	0.36	0.56	32	0.64	0.65
(5)	No one can help me.	1	0.33	0.58	28	0.72	0.71
(6)	I feel that I cannot help myself.	1	0.47	0.63	39	0.70	0.73
(7)	I feel hopeless.	1	0.29	0.53	24	0.79	0.78
(8)	I feel irritable.	2	0.63	0.66	52	0.58	0.56
(9)	I do not cope well with life.	2	0.29	0.51	27	0.73	0.78
(10)	I have a lot of regret about my life.	2	0.38	0.59	33	0.55	0.57
(11)	I tend to feel hurt easily.	2	0.55	0.64	47	0.66	0.65
(12)	I feel distressed about what is happening to me.	2	0.74	0.68	61	0.59	0.59
(13)	I am not a worthwhile person.	1	0.15	0.39	14	0.65	0.63
(14)	I would rather not be alive.	1	0.10	0.35	9	0.64	0.56
(15)	I feel quite isolated or alone.	2	0.27	0.53	23	0.62	0.68
(16)	I feel trapped by what is happening to me.	2	0.38	0.60	32	0.72	0.75

*M, mean; SD, standard deviation;%, percentage of patients who responded with at least “sometimes” to the item; r_it_ (sub), part-whole-corrected item-test correlation for the corresponding subscale; r_it_ (tot): part-whole-corrected item-test correlation for the total scale. 1, Meaning and Purpose subscale; 2, Distress and Coping Ability subscale.*

All part-whole-corrected item-test correlations were above 0.50, and the contributions of the items to the assigned subscale were about as high as the contributions to the total scale (Table 2).

The mean scores for the total scale were *M* = 5.78, *SD* = 6.34, and for the subscales *M* = 2.20, *SD* = 3.20 (*Meaning and Purpose)* and *M* = 3.58, *SD* = 3.45 (*Distress and Coping Ability*). Using the cutoff criteria for low (0–3), moderate (4–10) and high (≥11) demoralization, the percentages were 50.5% (low), 27.1% (moderate), and 22.4% (high demoralization).

### Test of the Dimensional Structure

Table 3 presents the results of the CFA. Both models, the one-factor model and the two-factor model, yielded similar CFA results, with CFI and TLI coefficients between 0.910 and 0.933 and SRMR coefficients <0.05. The correlation between the two subscales of the DS-II was *r* = 0.82.

**TABLE 3:** Summary of fit indices of the models.

	χ^ 2^	(df)	CMIN/DF	CFI	TLI	RMSEA	SRMR	BIC
1-factor model	443.3	(104)	4.26	0.922	0.910	0.077	0.044	10768
2-factor model	395.6	(103)	3.84	0.933	0.922	0.072	0.042	10727

*df, degrees of freedom; CMIN/DF, minimum discrepancy, divided by its degrees of freedom; CFI, Comparative Fit Index; TLI, Tucker-Lewis Index; RMSEA, Root Mean Square Error of Approximation; SRMR, Standardized Root Mean Squared Residual; BIC, Bayesian Information Criterion.*

### Convergent Validity of the Demoralization Scale-II With Other Measures of Psychological Distress

Table 4 presents associations between the scales of the DS-II and other scales measuring psychological distress. The correlations were highest for anxiety (GAD-2), followed by depression (PHQ-9), with coefficients of about *r* = 0.70, while the Distress Thermometer score was less strongly associated with demoralization (*r* = 0.36 for the total scale). In all cases, the correlations between the DS-II subscale *Distress and Coping Ability* and the other scales was higher than those with the subscale *Meaning and Purpose*.

**TABLE 4:** Correlations between demoralization and other variables of psychological distress.

	DS-II Total scale	DS-II Meaning and purpose	DS-II Distress and coping
PHQ-9: depression	0.69	0.63	0.68
GAD-2: anxiety	0.72	0.65	0.72
DT: mental distress	0.36	0.29	0.39
BIS: body image disturbance	0.58	0.52	0.58

*All Pearson correlation coefficients are statistically significant with p < 0.001.*

### Demoralization, Sociodemographic, Disease- and Treatment-Related Variables

The associations between demoralization, sociodemographic, disease- and treatment-related variables are presented in Table 5. Women (*M* = 5.97, *SD* = 6.20) reported slightly higher demoralization than men (*M* = 5.53, *SD* = 6.53) did, but the effect was far from being statistically significant. Concerning age, the highest demoralization was found in the youngest age group (18–49 years: *M* = 7.77, *SD* = 6.26), while the patients of the oldest age group (≥70 years: *M* = 4.44, *SD* = 5.65) were least demoralized.

**TABLE 5:** Demoralization Scale-II mean scores depending on sociodemographic, disease- and treatment-related variables.

	*n*	DS-II total	
		*M*	*SD*
**Gender^a^**	*F* = 0.013; *p* = 0.910		
Men	268	5.53	6.53
Women	352	5.97	6.20
**Age group^b^**	*F* = 6.924; *p* < 0.001		
≤49 years	133	7.77	6.26
60–59 years	144	5.51	6.09
60–69 years	172	5.81	6.90
≥70 years	171	4.44	5.65
**Employment status**	*F* = 2.403; *p* = 0.067		
Occupied	239	5.90	5.88
Unemployed	38	8.03	7.91
Retired	290	5.29	6.40
Other situation	29	7.90	7.87
**Civil status**	*F* = 5.922; *p* = 0.001		
Single	97	7.58	6.78
Married	372	4.80	5.68
Divorced/separated	97	7.64	7.29
Widowed	49	5.90	6.78
**Education**	*F* = 0.488; *p* = 0.485		
≤10 years of education	367	5.53	6.28
>10 years of education	244	6.20	6.41
**Tumor site**	*F* = 3.011; *p* = 0.002		
Breast	208	4.98	5.55
Prostate	104	4.75	7.08
Head and Neck	51	6.57	7.22
Skin	38	5.39	5.02
Female genital organs	34	7.32	7.05
Hematological	32	6.19	5.16
Central nervous system	30	5.67	5.49
Lung	28	9.29	8.20
Colorectal	22	8.23	6.31
**Metastases**	*F* = 0.352; *p* = 0.553		
No	477	5.56	6.19
Yes	110	6.04	6.16
**Surgery**	*F* = 1.527; *p* = 0.217		
No	140	5.99	6.71
Yes	465	5.69	6.17
**Chemotherapy**	*F* = 0.831; *p* = 0.326		
No	366	5.34	6.57
Yes	239	6.40	5.81
**Radiotherapy**	*F* = 13.664; *p* < 0.001		
No	175	7.46	6.60
Yes	432	5.05	6.03
**Hormone therapy**	*F* = 0.001; *p* = 0.980		
No	505	5.75	6.26
Yes	100	5.67	6.44

*Significance tests refer to ANOVAs with age group and gender as covariates; ^a^ controlled for age group; ^b^ controlled for gender.*

Including age and gender as covariates, ANOVAs revealed high demoralization mean scores for divorced/separated patients (*M* = 7.64, *SD* = 7.29). Patient with a diagnosis of lung cancer reported the highest rates of demoralization (*M* = 9.29, *SD* = 8.20), while patients with prostate cancer (*M* = 4.75, *SD* = 7.08) and patients with breast cancer (*M* = 4.98, *SD* = 5.55) being the least demoralized. Patients with metastases (*M* = 6.04, *SD* = 6.16) were not significantly less demoralized than patients without metastases (*M* = 5.56, *SD* = 6.19).

Treatment conditions were differently associated with demoralization: Patients who had not received radiotherapy reported significantly higher levels of demoralization (*M* = 7.46, *SD* = 6.60) compared to patients who received radiotherapy (*M* = 5.05, *SD* = 6.03), whereas we found no association between demoralization scores and having received surgery, chemotherapy, or hormone therapy.

## Discussion

The first aim of this study was to test the psychometric properties and reliability of the DS-II. All of the items contributed substantially to the total score and to the subscales, and the Cronbach‘s alpha coefficients for the total scale and for the subscales were almost excellent. The coefficients were slightly higher than in the study testing the DS-II in a sample of patients receiving palliative care (Robinson et al., 2016b), and they were also higher than the coefficients obtained in a sample of Spanish patients with advanced cancer (Belar et al., 2019). The higher reliability coefficients may be explained (at least partly) by the fact that our study did not only include patients with advanced stages of cancer, some of whom suffer from cognitive impairment, so coherent item responses may have been somewhat more likely in our sample. The mean score in our sample was lower than that of the sample in the original DS-II study with advanced cancer or other progressive diseases who were receiving palliative care (*M* = 7.64, *SD* = 6.43, Robinson et al., 2016b). Interestingly, for the DS-I with 24 items, the Cronbach‘s alpha coefficients of the total scale (α = 0.94), obtained in a general populations sample (Quintero Garzón et al., 2021), was nearly exactly as high as the coefficient (α = 0.93) in our sample.

However, the CFA results for both the one-factor solution and the two-factor solution failed to meet the common criteria for good model fit. Only the SRMR coefficient was within the range of good model fit for both factorial solutions. This means that there is some structure in the items that is not explained by the models. We could have defined factors that would be better suited to our own data set as has been done in previous research on the DS-I scale, however, this would probably not contribute to the comparability of international studies on demoralization. To omit problems with the subscales we believe that, due to the high Cronbach‘s alpha coefficient, it is at least justified to consider the one-dimensional solution of the DS-II.

As expected from previous studies, we also found a strong correlation between demoralization and depression, which demonstrated a close relationship between these concepts. This association is about as high as that reported for outpatients in a psychiatric oncology clinic (Ignatius and De La Garza, 2019) and the relationship between demoralization and depression in the Spanish sample of patients with advanced cancer (Belar et al., 2019). A lower correlation between DS-II and the PHQ-9 score was found for the patients receiving palliative care (*r* = 0.41) in the study introducing the DS-II (Robinson et al., 2016b), however, in that calculation, the PHQ-9 was only used in two categories.

Despite the positive association between depression and demoralization, both constructs can be distinguished by its core symptoms (depression: loss of pleasure and interest vs. demoralization: a loss of meaning, Robinson et al., 2015) and a dimensional characterization of depressed/demoralized patients indicates possible implications for treatment (Belvederi Murri et al., 2020a). Recent research identified a proportion of patients [17% (Wu et al., 2021) up to 28% (Tang et al., 2020)] with high levels of demoralization but low depression scores to whom special attention must be paid in clinical care. It is possible that these patients will be less likely to respond to antidepressants and are likely to respond best to meaning-centered psychotherapies (Ignatius and De La Garza, 2019).

The association between demoralization and anxiety was even higher than that with depression in our sample. This finding is in line with previous research demonstrating that demoralization is closely linked to the construct of anxiety (Mehnert et al., 2011; Nanni et al., 2018), and especially death anxiety in patients with advanced cancer (An et al., 2018). Anxiety disorders occur in comorbidity with demoralization in about a quarter of medically ill patients, particularly, generalized anxiety disorder, agoraphobia, and panic disorder (Rafanelli et al., 2013). The results also showed a significant association between demoralization and body image disturbance indicating that body-related issues should be addressed and considered in the psychosocial treatment of patients reporting demoralization.

We found a relevant decrease in demoralization with increasing age. This negative correlation confirms the results of two previous studies (Mehnert et al., 2011; Vehling et al., 2013) and was also reported for the *Distress and Coping Ability* subscale (Robinson et al., 2016a). In addition, multiple studies on depression and anxiety in patients with cancer have shown that, compared with the general population, young patients with cancer are much more affected than older ones (Götze et al., 2020). There are no normative values that could be used for such comparisons for the DS-II. A recent normative study with the DS-I failed to find a linear association between age and demoralization (Quintero Garzón et al., 2021); the highest scores were observed for the age categories <30 years, 50–59 years, and ≥70 years. Because of the lack of normative values for the DS-II scale we cannot assess the extent to which the age effect found in our study is specific for patients with cancer.

In accordance with previous evidence we found no association between demoralization and gender (Robinson et al., 2016a), while in the general population, demoralization (as measured with the DS-I) is higher in females than in males, with an effect size of *d* = 0.12 (Quintero Garzón et al., 2021).

Our results showed that being divorced/separated is related to higher levels of demoralization, a finding that is in line with the results of four previous studies (Katz et al., 2001; Grandi et al., 2011; Mehnert et al., 2011; Robinson et al., 2016a) and confirms the inverse relation between social support and demoralization.

Our results showed that the presence of metastases had no significant impact on demoralization. This is unlikely to be an indirect age effect since both groups, those with and without metastases, were of similar age (59.2 and 60.5 years, respectively). In addition, this finding is consistent with four other studies showing no association between demoralization and stage of disease (Grassi et al., 2004; Boscaglia and Clarke, 2007; Lee et al., 2012; Vehling et al., 2012). However, we found a significant association between demoralization and tumor site (highest demoralization scores for patients with lung cancer), which has not been reported in previous studies (Mehnert et al., 2011; Robinson et al., 2016a). Moreover, in contrast to previous findings (Robinson et al., 2016a) we obtained a significant association between cancer-specific treatment and demoralization, with significantly higher demoralization scores for patients who had not received radiotherapy compared to patients who had received radiotherapy. However, the lower demoralization scores in patients receiving radiotherapy may be confounded with tumor site. These findings add to the mixed evidence related to the association between demoralization and disease- and treatment-related variables and should be further investigated in future studies.

Several limitations of this study should be mentioned. Most of the patients were recruited in a counseling center. This sample may not be representative of all patients with cancer. These are patients who both perceive a need for help and are able to actively seek professional support. Therefore, we cannot conclude that it is a sample with especially high or especially low levels of burden. In addition, until now, no normative study has been conducted with the DS-II; therefore, the interpretability of the mean scores is limited. In particular, it would be relevant to examine whether age and gender differences obtained in patients with cancer can also be found in general population samples. Furthermore, according to our study design, we are not able to calculate test-retest reliability and sensitivity to change scores.

For future studies, it would be worthwhile to investigate how the DS-II can be used in clinical contexts in which patients do not experience psychological distress associated with cancer, but may still experience feelings of incompetence, and feelings of helplessness and hopelessness in response to a stressful life event. For example, demoralization was assessed with the DS-II scale in a sample of 209 postnatal women admitted with their babies to a residential early parenting program (Bobevski et al., 2018). The authors derived a 14-item revised scale, the Postnatal DS-II, showing good psychometric properties (Bobevski et al., 2018). Moreover, in a recent study with medically ill patients in general hospitals, even shorter versions of the Demoralization Scale with 13- and 6-items were developed, with both versions retaining high correlations with DS-I scores and concordance with the Diagnostic Criteria for Psychosomatic Research-Demoralization module (Belvederi Murri et al., 2020b).

From a clinical perspective, demoralization is a treatable condition and its diagnosis may empower clinicians to provide proper psychological care, something which is especially true with regard to the strong correlation between demoralization and suicidal ideation (Costanza et al., 2020). In this context, it is highly relevant to have access to a short, user-friendly and psychometrically sound measure of demoralization to correctly classify and distinguish the patients’ emotional states. Using the DS-II may guide clinicians’ efforts to provide appropriate interventions as early as possible, e.g., refer patients with high demoralization scores to meaning-centered interventions in order to enhance meaning and purpose and to promote coping abilities while facing existential threats (Breitbart et al., 2015, 2018). The DS-II scale can be easily incorporated into psychosocial, clinical, and palliative care settings as a brief screening measure to identify patients with high levels of demoralization and severe existential distress.

In summary, our study translated the DS-II into German and tested its psychometric properties. The psychometric evaluation demonstrated that the instrument shows satisfactory findings concerning the assessment of demoralization in patients with cancer. Further studies are needed to determine the best item structure of the DS-II scale and to gather normative data to put values from patients with cancer into context. Furthermore, we conclude that the results support the implementation of regular screening for demoralization in certain risk groups, as demoralization scores are significantly increased in patients at a younger age, divorced/separated patients, patients with a lung cancer diagnosis and in patients receiving no radiotherapy.

## Data Availability Statement

The raw data supporting the conclusions of this article will be made available by the authors, without undue reservation.

## Ethics Statement

The studies involving human participants were reviewed and approved by the Ethics Committee of the University of Leipzig. The patients/participants provided their written informed consent to participate in this study.

## Author Contributions

SK, AH, and AM-T contributed to the conception and design. AM-T, UF, AL, and MR provided the administrative support. TH, AM-T, UF, AL, and MR contributed to the provision of study materials and patients. TH contributed to the collection and assembly of data. SK, AH, JH, LQG, and PE contributed to the data analysis and interpretation. SK and AH were mainly responsible for writing the manuscript, and all authors gave final approval of the manuscript.

## Conflict of Interest

The authors declare that the research was conducted in the absence of any commercial or financial relationships that could be construed as a potential conflict of interest.

## Publisher’s Note

All claims expressed in this article are solely those of the authors and do not necessarily represent those of their affiliated organizations, or those of the publisher, the editors and the reviewers. Any product that may be evaluated in this article, or claim that may be made by its manufacturer, is not guaranteed or endorsed by the publisher.
